# Integrating Public Health into Climate Change Policy and Planning: State of Practice Update

**DOI:** 10.3390/ijerph16183232

**Published:** 2019-09-04

**Authors:** Mary Fox, Christopher Zuidema, Bridget Bauman, Thomas Burke, Mary Sheehan

**Affiliations:** 1 Department of Health Policy and Management, Risk Sciences and Public Policy Institute, Johns Hopkins Bloomberg School of Public Health, Baltimore, MD 21205, USA (T.B.) (M.S.); 2 Department of Environmental Health and Engineering, Johns Hopkins Bloomberg School of Public Health, Baltimore, MD 21205, USA (C.Z.) (B.B.)

**Keywords:** adaptation, adaptive management, climate change, essential services of public health, governance, implementation, mitigation, public health practice

## Abstract

Policy action in the coming decade will be crucial to achieving globally agreed upon goals to decarbonize the economy and build resilience to a warmer, more extreme climate. Public health has an essential role in climate planning and action: “Co-benefits” to health help underpin greenhouse gas reduction strategies, while safeguarding health—particularly of the most vulnerable—is a frontline local adaptation goal. Using the structure of the core functions and essential services (CFES), we reviewed the literature documenting the evolution of public health’s role in climate change action since the 2009 launch of the US CDC Climate and Health Program. We found that the public health response to climate change has been promising in the area of assessment (monitoring climate hazards, diagnosing health status, assessing vulnerability); mixed in the area of policy development (mobilizing partnerships, mitigation and adaptation activities); and relatively weak in assurance (communication, workforce development and evaluation). We suggest that the CFES model remains important, but is not aligned with three concepts—governance, implementation and adjustment—that have taken on increasing importance. Adding these concepts to the model can help ensure that public health fulfills its potential as a proactive partner fully integrated into climate policy planning and action in the coming decade.

## 1. Introduction

With accelerating frequency and intensity, severe storms, heatwaves, wildfires, droughts and other extreme weather events are having ever-more evident impacts on human health and wellbeing. Among these impacts are heat-related illness, injuries and losses due to flooding; exacerbation of asthma, respiratory and cardiovascular conditions with air pollution; and growing risks of vector-borne diseases (malaria, dengue, Lyme disease); water-borne diseases (diarrhea, cholera) and chemical pollutants; undernutrition; and forced migration [1,2]. These many impacts are unlikely to occur in isolation; combined and cumulative effects are likely. Research now indicates that many specific extreme weather events have been made more likely by global warming [3]. Recent record-breaking heat and precipitation and their impacts on human populations—e.g., 2017′s unprecedented Atlantic hurricane season, or the deadly complex emergency of California wildfires and devastating heatwaves and flooding in India and Pakistan in 2018—led one veteran climate scientist to suggest that 2018 was the year the world woke up to climate change “not as a problem for future generations, but for us now” [4]. Also in 2018, the Intergovernmental Panel on Climate Change (IPCC) issued its stark report Global Warming of 1.5 Degrees, which makes clear the substantially higher risks to human populations of 2.0 °C compared to 1.5 °C of additional warming (the aspirational target of the 2015 Paris Agreement), and warns of the need for “rapid and far-reaching transitions in land, urban, infrastructure, and industrial systems,” including ambitious negative emissions targets [5]. IPCC modeling indicates Earth may reach 1.5 °C of additional warming as early as 2030. Therefore, the coming ten-year period will be crucial for society’s dual challenges of decarbonizing the economy and preparing for more extreme climate conditions. Public health has a key role to play and must take on greater leadership in the decade ahead to achieve both of these goals.

While the causes of climate change are global, health impacts are inherently local—they happen where people live, work, learn, play and travel. Due to geography, exposure, and sensitivity to health effects, some local populations are substantially more vulnerable than others [6]. For example, cities are often on the front-line of climate impacts due to their densely concentrated populations, the urban heat island effect which can make them substantially warmer than outlying regions, their frequent proximity to coasts and waterways, and reliance on ageing physical infrastructure networks [7]. Local sub-national governments in many regions have been initiators of climate change policy due to this proximity to impacts, as well as slower national-level action on climate policy in some cases [8]. Public planning and policy-making for climate change has appropriately focused on reducing greenhouse gas (GHG) emissions generated by energy, transport, industry and land use/agriculture, often leaving public health somewhat on the margins of climate action planning [9,10,11]. However, because the goal of much climate-adaptive policy is to protect human wellbeing at the local level where impacts occur, public health is a natural leader of adaptation efforts [2]. In addition, as noted by Woodward and Samet, “the public health consequences of climate change are a critical element of the rationale for [climate] action,” making them a potential driver of GHG reductions as well [8].

More recently, the public health field has begun to assume higher visibility in climate change policy. The World Health Organization (WHO) called the Paris Agreement “a fundamental public health agreement” [12], and major reports such as the Lancet Countdown: Tracking Progress on Health and Climate Change [2,13] have made clear the central role of the health field in both identifying and communicating economic health “co-benefits” from reduced GHGs, and contributing to the widespread need to target vulnerability and build local resilience. Public health is thus increasingly recognized as a key player in the twin tasks of reducing GHG emissions and adapting to a warmer, less predictable climate [2,8]. Nevertheless, multiple challenges remain for public health to fully live up to its potential leadership role [14,15].

A decade ago, scientists from the US Centers for Disease Control and Prevention (CDC) published an influential paper laying out the conceptual and ethical rationale for a public health approach to climate change based on the ten “essential public health services” (Box 1) that guide and inform the carrying out of public health practice [16]. That analysis identified numerous ways in which ongoing public health activities were already supporting climate change-related policy-making, suggested activities new to the public health field that were emerging as a result of the challenge of climate change, and proposed gaps and needs to safeguard public health in light of a warming and more unpredictable future climate. These authors concluded that many existing public health responses could effectively address emerging climate change impacts, but considered in particular that enhanced coordination across government agencies and between government and non-government actors was needed.

Box 1 **Public health core functions and essential services.**

**Figure 1:**
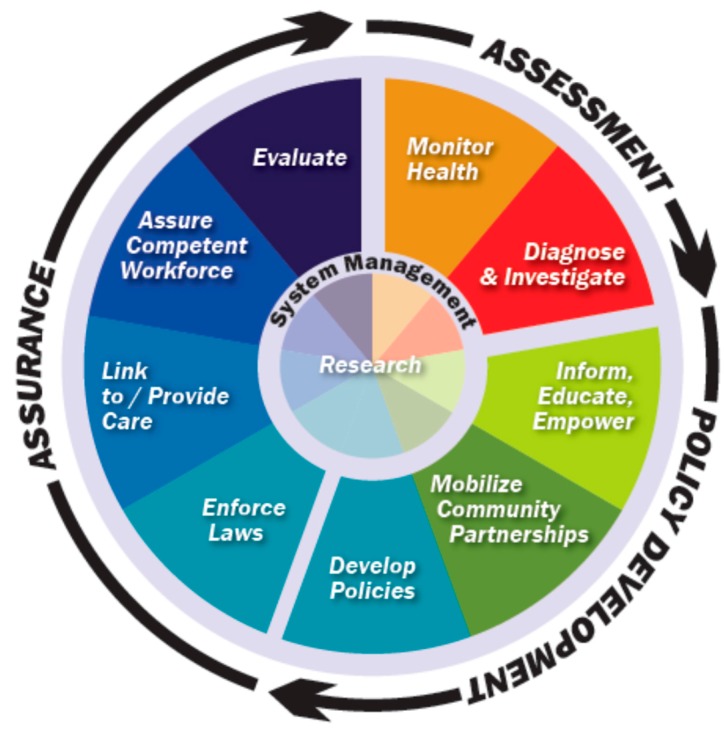
The core functions and essential services (CFES) model [24].

The three core functions of public health—assessment, policy development, and assurance—were originally recommended by an Institute of Medicine (IOM) committee as a way to organize public health activities [17]. Efforts to operationalize these core functions occurred in the following years, resulting in the 1994 “Public Health in America” statement, and development of the ten essential public health services. These are to: monitor health; investigate and diagnose; educate, inform and empower; mobilize partnerships; develop policies; enforce laws and regulations; provide and link to care; assure a competent workforce; evaluate; and research [18,19]. The ten essential services elucidate specific activities corresponding to each core function, as shown schematically in Figure 1.

The ten essential services, as organized by the core public health functions of assessment, policy development and assurance, also served as underpinning to CDC’s nation-wide Climate and Health Program (CHP), launched in 2009. Designed to support local government efforts toward climate adaptation, CHP developed new tools, including the “Building Resilience Against Climate Effects” (BRACE) assessment framework for sub-national governments implemented through the Climate Ready States and Cities Initiative [20,21]. Numerous US states and cities have successfully used BRACE to enhance their climate and health adaptation activities [22]. This “core functions and essential services (CFES) model” (Figure 1) relied upon by the CHP has continued to be employed as a guide to developing policy and practice responses for the challenges of climate change, e.g., in the call by Wheeler and Watts to translate climate science into public health practice [23]. 

### Approach 

This narrative review examines progress toward implementing a public health approach to climate change by carrying out a ten-year update on the analysis underpinning the CHP [16] using the structure of the CFES model. Specifically, we aim to identify evidence of forward policy and practice movement through use of specific climate and health tools in each of the ten essential public health service areas.

To achieve these aims, we carried out a “review of reviews” of research in each of the ten essential service areas. We performed a targeted search of the English-language literature published between end-2008 and end-2018 to capture developments after publication of the CDC work mentioned above. Both PubMed and Web of Science were searched to identify relevant climate and health and climate science and health literatures, respectively. Search terms were related to each of the essential services as well as terms for climate change and health. We also scanned bibliographies of recent reviews for relevant papers and agency reports. Given the large literature available, we limited inclusion in our synthesis to review papers; however, when no (or limited) reviews were available we relied upon recent non-review articles. Separately, we identified climate and health practice examples using online sources.

We provide in Results below a narrative synthesis of findings, highlighting a selection of major themes in the evolution of practice over the last decade, organized by core function and essential service. In the process of review and synthesis, we also examined the fit of the CFES model to the public health-related risks and opportunities of climate change based on evolving knowledge, elaborated in the Discussion section. 

## 2. Results

### 2.1. Assessment 

Public health assessment entails collecting data, and analyzing, investigating and identifying public health problems; it informs policy development and sets the stage for public health assurance, the second and third of the core functions [17]. When integrated with the ten essential public health services using a climate change lens, assessment involves (i): monitoring health status with regard to climate-sensitive outcomes, and (ii) investigation and diagnosis of health impacts linked to climate-related hazards (Figure 1). 

#### 2.1.1. Monitor Health

Frumkin et al. identified three categories of data needed to monitor health status in the context of a changing climate: (i) environmental risk or exposure (e.g., climate conditions); (ii) population vulnerability (e.g., geographic location); and (iii) disease-related surveillance (e.g., climate-sensitive diseases) [16]. Since then, the literature suggests that there has been progress in each of these areas.

##### Exposure to Climate Hazards

Recognition of the need for appropriately scaled climate forecasts to determine exposure to climate hazards was a key data concern a decade ago. Readily accessible “climate services” information—that is, climate and weather data along with their interpretation and downscaling to the local level—are essential to developing preventive climate-health policy responses. A major development toward this goal was creation in 2012 of the Global Framework for Climate Services (GFCS) led by the World Meteorological Organization (WMO); GFCS aims to improve meteorological information and targets public health among its key priorities [25]. Jancloes et al. noted progress in enhancing spatiotemporal resolution of this type of climate data, for example making it more relevant for weather-hazard early warning efforts (see Enforce Laws and Regulations, below), and more tangible for the public [26]. Georgeson et al. quantified the global commercial supply of weather-related information services and found overall improvement of these services internationally over the last years [27].

However, despite increasing supply of climate services information, access across countries has been found to be uneven [27]. Spatial scale remains challenging; for example, under the CDC CHP program, localities often required technical partners to assist with downscaling data to the locally relevant level [22]. Promising initiatives such as the Climate Data Factory [28]—which provides web-based downscaled climate information on a commercial basis for over 4300 cities in 70 countries—may help to close information gaps at the local level over time. 

##### Vulnerability

Identifying population vulnerability is a key component of assessment frameworks supporting climate change adaptation (see Investigate and Diagnose, below). Since 2008, recognition of the importance of vulnerability factors and how they differ across populations has increased; one author referred to a “new landscape of inequality” [29]. For example, English and Richardson reviewed vulnerability factors affecting climate adaptation and flagged urban populations in the fast-growing cities of Asia and Africa as among those at greatest risk [30]. The multi-sectoral and inequitable nature of health risks contributes, along with other factors, to complexity in identifying climate-health adaptation policy solutions, requiring new and population-specific approaches [31]. 

A decade ago, vulnerability indices for flood risk were flagged as a key tool for targeting those at greatest risk. Since that time, the literature reflects broader use of such indices. For example, the Social Vulnerability Index (SVI) developed by the US CDC (based on census data for factors such as socioeconomic status and available infrastructure), has been adjusted for climate-specific risks; linkage to GIS mapping helps to visualize climate-related health risks and can be used to target emergency response [32,33]. Other hazard-specific indices and their geospatial mapping have increasingly been integrated into adaptation planning toolkits, particularly for flooding and extreme heat. A good practice example is the San Francisco health department’s heat vulnerability index (HVI), which identifies and maps six factors (socioeconomic status and isolation, air quality, urban density, lack of vegetation and being elderly) responsible for 70% of variability in risk of heat-related illness [34]. The Notre Dame Global Adaptation Initiative (ND-GAIN) index of 278 US cities—which covers flooding, heat, cold, drought and sea-level risk—is an example of a broader “climate readiness” index using similar methods [35]. 

Research suggests that enhancements are warranted in vulnerability targeting tools, however. Preston et al. reviewed 45 climate vulnerability index studies of various sorts, finding diversity in framing of determinants and suggesting greater clarity was needed regarding the goals of these tools, and encouraging “capitalizing on the power of maps” to more systematically visually engage stakeholders [36]. A review of 15 heat vulnerability index studies concluded that the HVI is useful for targeting interventions, though recommended improved definition of heat-related indicators and their appropriate weighting to optimize HVI use [37]. Other research validated five commonly used natural-disaster vulnerability indices against data in the US southeast, however found that they performed less well explaining human fatalities than economic damage [38].

##### Disease Surveillance

Surveillance for infectious diseases is part of the standard public health toolkit. A decade ago, the challenge was seen as strengthening non-infectious climate-sensitive disease surveillance while harmonizing collection of exposure, vulnerability and surveillance data at similar temporal and spatial scales to support climate-adaptive responses. Modifying existing systems, such as the US National Environmental Public Health Tracking (EPHT) Program was seen as a promising approach [16]. Since then, several initiatives toward harmonization of climate and health monitoring and surveillance systems have emerged. The climate focus of the EPHT Program has been revised to include twelve climate exposure indicators (including temperature distribution, historical heat and extreme precipitation data, and future heat and precipitation predictions); flood and heat vulnerability indicators; and health outcomes data [39]. Moulton and Schramm [40] observed valuable progress across independent efforts by agencies at national and local levels in the US, while Houghton and English [41] proposed a harmonized three-tiered approach to developing climate and health indicators that builds on such local surveillance. Other efforts have focused on targeted surveillance for specific diseases toward development of early warning systems (see Enforce Laws and Regulations, below), for example dengue fever in the European Union [42]. At the local level, the New Jersey Department of Health developed the first use of syndromic surveillance software to monitor severe weather-related outcomes such as carbon monoxide poisoning, anxiety and adjustment disorders, and disrupted outpatient medical care in the wake of Hurricane Sandy [43]. 

At a global level, The Lancet Countdown on Health and Climate Change, a collaborative effort of 27 universities and international agencies, launched a set of 41 indicators (to be updated over time) that offer an independent monitoring system for comprehensive data collection on health and climate change across five thematic areas: climate change impacts, exposures and vulnerability; adaptation, planning and resilience for health; mitigation actions and health co-benefits; finance and economics; and public and political engagement [2,13]. The goal of this annual effort is to track world progress on the health response to climate change. Findings of the 2018 report highlight global trends, including emerging extreme heat-related risks, the vulnerability of health systems, progress toward low-carbon transition, and the potential for public health to be “vital in delivering an accelerated response” to climate change [2]. The Lancet indicators are likely to be influential over time in shaping the monitoring systems of national and local public health agencies whose data feed into this system.

Nevertheless, despite this substantial progress, effective and harmonized climate-health surveillance remains a challenge. Moulton and Schramm [40] point out that US national and sub-national surveillance initiatives lack a shared conceptual framework and have focused mainly on indicator and data source development to the exclusion of systems and tools, capacity building, and supportive policies. At the global level, Ebi et al. [44] point out the need for indicators of impacts and resilience of health systems, and the need for indicators that dynamically reflect changing risks and emerging adaptive solutions. Similarly, Erwin and Brownson [45] recommended a specific “policy” indicator for tracking the process, content, and outcomes of largescale changes. Important disease-specific data challenges also remain; for example, while monitoring of heat-related illness (HRI) has improved, Morano et al. identified the need to enhance ICD injury diagnosis and codes to improve HRI surveillance sensitivity [46].

#### 2.1.2. Investigate and Diagnose

Ten years ago, the long-established public health roles of outbreak investigation and intervention were seen as critical in responding to the changing climate, while fine-tuning risk assessment methods was considered a pathway to addressing the challenge of attributing health outcomes to climate-related exposures and estimating burden of disease [16]. Among key developments over the decade has been greater recognition of the dynamic nature of climate change risks to health, resulting in modifications to risk assessment frameworks to reflect complex systems thinking and Bayesian network approaches [47,48,49,50]. Another theme of assessment research has been the limitation imposed by the uncertain nature of the data used to predict health outcomes, related not only to climate parameters but also to demographic and socio-economic conditions, and adaptive factors such as financial and institutional capacity and community-level social capital [48,51,52]. Data and model uncertainty are ubiquitous challenges in the development of complex assessments in any context; as new data become available and experience is gained over time, uncertainty will be reduced. The public health challenge then becomes translating advances in knowledge from improved data and models into practical responses. Hess et al. [52] recommended an approach that allows for dynamic adjustment to climate change hazards under conditions of uncertainty by incorporating learning into climate-related risk assessment. This “adaptive management” cycle starts with assessment, continues with planning, implementing, monitoring, evaluation, and adjustment, and then returns to assessment [53]. The CDC BRACE framework [21] uses this iterative approach, as does the EU Climate ADAPT framework [54] and others. The Hess et al. adaptive management model evokes the cyclical nature of the core functions and essential services of public health model, but adds an adjustment step to reflect this dynamic uncertainty [52]. 

Health Impact Assessment (HIA) has become an additional assessment tool to identify climate hazards and risks to health due to projects and programs being implemented across a range of non-health sectors. HIA’s five-step process—screening, scoping, appraisal, reporting, and monitoring [55]—is a flexible, multi-disciplinary and collaborative approach involving the input and expertise of a range of public health and other practitioners, officials, and community participants [56]. A core tool of the Health in All Policies (HIAP) approach—increasingly used in many countries to assess the multi-sectoral nature of health impacts from climate hazards [57]—HIA can be applied to anticipate how climate change will impact communities [56,58], as well as to identify ways mitigation or adaptation activities may result in co-benefits to population health (see Policy Development, mitigation policy, below). Looking ahead, further development and use of the multi-sectoral approach encouraged by HIA represents an opportunity to identify tradeoffs and synergies in policy options (see Policy Development, mobilize partnerships, below).

### 2.2. Policy Development 

Policy development is the second phase of the core functions cycle (Figure 1); it builds on the diagnosis from assessment, and encompasses identification and planning of actions that will be carried out and monitored in the assurance phase. Policy development involves not only government efforts to establish laws, regulations and procedures but also voluntary practices of private actors and efforts to communicate with the public. When integrated with the ten essential public health services in the context of climate change, policy development covers: (i) informing, educating and empowering populations toward climate resilience; (ii) mobilizing partnerships to anticipate and respond to health threats from climate change; and (iii) development of health policies that support climate mitigation and adaptation.

#### 2.2.1. Inform, Educate, Empower

The challenges identified a decade ago were to take into account varying levels of climate-related understanding in the population, emphasize constructive health behaviors, and evaluate communication interventions [16]. Evidence over the last decade suggests understanding of climate change and impacts on health remains low in a range of populations in the US [59,60], including among health professionals—of particular concern considering research indicating the public trusts first and foremost their primary health care providers for such information [61]. However, both the general public and health professionals appear to express strong openness to learning more about health impacts of climate change [60]. Ziegler et al. offer guidance to physicians, including advocacy for adaptation and mitigation strategies for the populations they serve [62]. Meanwhile, research on climate change and health risk education confirms its greater effectiveness when paired with concrete suggestions for action [63,64]. 

Health communications research has also provided useful insights for simplifying, translating and reframing scientific messages about climate change and health [65,66]. Depoux et al. note that the “scientific voice” does not effectively convey climate change and health messages, and recommend public health framing with related behavior change action items [64]. Dervin and Rudolf recommend modeling on successful public health campaigns such as tobacco [67]. Targeted reframing may also assist in message uptake for certain groups; for example, Zia and Todd suggest that among those with conservative ideology, positioning health impacts of climate change as an economic or security issue may increase effectiveness [68]. Engaging prominent popular culture or other leaders as “champions” to deliver climate health messages has also been suggested. For example, using “big data” analytics Leas et al. analyzed social media references to “climate change” and found that Leonardo DiCaprio’s 2016 Oscar acceptance speech surpassed the daily average effect of the 2015 Paris Agreement [69]. 

#### 2.2.2. Mobilize Partnerships

In 2008, the public health approach to climate change recommended was one that “emphasizes the coordination of government agencies (federal, state, and local), academia, the private sector, and nongovernmental organizations” [16]. The literature in the last ten years continues to reflect the need for collaboration across sectors and disciplines [10,70]. This is reflected in an enhanced attention to governance—or, the ways in which authority and resources are allocated for coordinated policy efforts [70,71]—and how this translates into partnerships. Austin et al. suggest three areas of partnership for health and climate governance: (i) coordination across different levels of government on health monitoring, diagnosis and policy implementation; (ii) collaboration of public health with non-health sectors, particularly to share health-related epidemiological, vulnerability, program monitoring and evaluation data; and (iii) partnerships with private and non-governmental organizations to implement programs to achieve specific health outcomes [14]. The literature suggests developments in all three areas over the last decade.

##### Coordinate across Government Levels

Governments at both national and subnational level play a key role in public health climate policy, with the most commonly employed tool being climate adaptation planning. In their review of ten OECD health adaptation efforts, Austin et al., found that the role of national governments in health adaptation centered largely on indicating policy priorities, research, funding, coordination and guidance for population-level initiatives; examples cited were Switzerland, Belgium, and the US, which have national-level programs [71]. Local government actions were more targeted and health-risk specific. These authors found that specific vertical coordination arrangements among national and subnational governments included institutionalized reporting and monitoring as well as informal communication channels, while national adaptation planning, coordinated by a climate change commission or an ad hoc working group, was seen as one way to build-in both horizontal cross-sectoral collaboration and vertical coordination among government levels [14].

##### Collaborate across Public Health and Non-Health Sectors

Research over the last decade emphasizes that climate-related health outcomes are often affected (and sometimes even determined) by service quality in sectors other than health [72]. In particular, malfunctions of traditional built environment infrastructure (e.g., energy, transport, water and sanitation, and urban planning) can be particularly challenging through the cascading effects of one service on another, potentially causing considerable and often longer-term wellbeing impacts [73]. For example, electric power outages in New York City during Hurricane Sandy in 2012 contributed to population health impacts through a variety of pathways, including curtailed hospital services, need to evacuate patients, inoperable pharmacies, and carbon monoxide poisoning with increased diesel generator use [74]. Bowen and Ebi suggest that creating networks, integration across organizations and jointly developed policies are some ways cross-sectoral collaboration can be carried out [75]. Patz et al. point out that such interdisciplinary collaboration is essential to addressing complexity in health and climate challenges [76]. In a survey of over 300 world cities preparing climate action plans, Aylett found that most worked to break down sectoral silos through informal communication channels and formal institutions such as inter-departmental working groups [10]. McCarney et al. found that shifting climate change out of environment departments where it has typically been housed was also a key local strategy [77]. However, of concern Aylett found that health was among several “marginalized” sectors which contributed least to broader city climate adaptation efforts [10].

##### Non-Government Partnerships

While governments have a primary role in developing policy that is protective of public health in the context of climate change, multiple reviews observed the critical role non-governmental organizations play in health adaptation [78,79]. Bowen et al. identified four “governance elements” that could be helpful in creating the appropriate environment for climate and health adaptation among non-government actors: social capital; non-state-based actors; informal networks, and bridging organizations [72]. An example of a bridging organization’s work is the Rockefeller Foundation’s establishment of the Asian Cities Climate Change Resilience network which is beginning to demonstrate results helping health systems to become more climate resilient [29]. In terms of non-state-based actors, the US Climate Resilience Toolkit is a source of multiple examples, including a non-profit group helping design a solar-powered food storage facility for a community in Alaska; and students in Georgia contributing to flood resilience by mapping the flood plain, analyzing flood water, making water filtration kits and developing messages for a communication campaign [80,81]. 

#### 2.2.3. Develop Policies

A decade ago, public health was seen as having a role in both explaining the rationale for climate mitigation through reduced health risks and providing evidence for health co-benefits and climate adaptation planning [16]. Since that time, the literature suggests that public health has taken a more proactive role in both of these policy development areas as well as in creating the conditions for policy development, i.e., building collaborations, engagement of communities and diverse stakeholders in discussion of needs and preferred policy options.

##### Mitigation

Substantial research over the decade has involved modeling and quantifying expected economic benefits related to population health from policies to reduce GHGs, particularly from power generation, transport, agriculture, household and industrial energy sectors [82]. For example, a global analysis found health benefits from reduced air pollution alone exceeded costs of reaching the Paris Agreement targets [83]. Work examining energy supply and efficiency, active-transport and dietary change related co-benefits at regional, national and city level suggested that health benefits are likely to be important particularly for developing countries [84,85,86,87]. However, Workman et al. noted that health has been “elusive in its influence” on climate mitigation policy due to vested economic interests and structural political issues; to enhance impact of health on mitigation policy these authors recommend better aligning health co-benefits with renewable energy goals, identifying visible champions, and emphasizing health benefits in communications [15]. Reflecting the broader priority to identifying co-benefits—and making the link between health co-benefits and renewable energy—the Lancet Countdown included nine mitigation indicators in its list of global measures, including zero-carbon emissions electricity, exposure to ambient air pollution in cities, and premature mortality from air pollution by sector [2]. 

HIA has increasingly been employed to identify and quantify co-benefits [56,88]. An HIA with an adaptive system-thinking methodology and a wide range of stakeholder’s input can help explain linkages between climate change and health and elucidate co-benefits [56,88,89,90]. HIA results can be helpful for adaptive management; e.g., modeling of co-benefits can create quantifiable measures to inform policy makers of the results of policy efforts through monitoring and evaluation [89,90,91,92]. However, because HIAs are designed for analysis of a program or policy in a particular place with extensive stakeholder engagement they therefore can be time consuming, data- and resource-intensive and can have results that vary from place to place [13,90,91]. 

##### Adaptation

Progress toward health adaptation planning has progressed from an aspirational goal to accomplishments over the decade and effective adaptation strategies are emerging, particularly for heat and vector-borne diseases, as further discussed below. Berry et al. reviewed vulnerability and adaptation assessments internationally, and found that these assessments had helped raise awareness, identify risks, build capacity and bring health into broader climate adaptation efforts [6]. However, they also noted that vulnerability was often overlooked, epidemiological data and climate scenarios were often lacking, and health authorities often have difficulty translating findings into implementable practice, including due to limited funding and capacity [6]. A review of the CDC’s CHP program similarly found that some sub-national public health department grantees benefited through the program by gaining a seat at the broader climate table, however, not all were able to do so [22]. 

Some adjustments are needed to adaptation policy planning. For example, Hayes and Poland (2018) argue in particular for including mental health concepts more explicitly in health vulnerability and adaptation assessments [93]. Araos et al. observe in their study of large cities that adaptation activities rarely addressed capacity building or research, and were lacking in monitoring, reporting, and evaluation components [11]. Banwell et al. make the case that vulnerability and adaptation assessments are an opportunity to synergistically link disaster risk reduction and adaptation—through commonalities such as vulnerability targeting or use of early warning systems—and that public health has a key role in this important connection [94]. However, Runckle et al. note that the public health community generally is unaware of the climate-related epidemiological and assessment tools available, in part due to lack of funding [95]. Reflecting some of these findings, the Lancet Countdown identified eight adaptation-related indicators for global monitoring including the number of national vulnerability and adaptation assessments, national adaptation plans for health, and city climate risk assessments, as well as amount of adaptation funding [2].

### 2.3. Assurance 

Assurance is the third phase of the core functions cycle (Figure 1); it is an implementation phase that grows out of policy development, where policies are put in to practice through regulations, programs, workforce training, and other activities. Assurance also completes the direct feedback loop through evaluation to the monitoring and diagnosis done under the assessment phase. This latter link provides the iterative opportunity for dynamic adjustment—needed for integrative, adaptive management in the complex context of climate change and human health. When integrated with the ten essential public health services from a climate-risk perspective, assurance involves: (i) enforcing laws related to climate-susceptible diseases and impacts; (ii) linking to and providing care in the context of climate hazards; (iii) assuring a competent workforce to respond to climate-related health threats; and (iv) evaluating climate health interventions.

#### 2.3.1. Enforce Laws and Regulations

In 2008, Frumkin et al. wrote that “few public health laws and regulations have a direct bearing on climate change [16].” That is now changing, as global warming and extreme weather have become a more pressing reality over the last decade. Public health agencies have traditionally enforced laws, regulations and other policy guidelines to protect population health. Important in the context of climate change, for example, are enforcement of safety regulations that protect from food- and water-borne disease (through restaurant licensing, food inspection and water sampling and testing); vector-borne disease management (through spraying to control mosquito populations). Risk of climate-sensitive diseases is projected to increase, with concurrent demand for enhanced public health services [96]. Increasing availability of climate services and modeling of relationships between weather parameters and disease (see Monitor Health, above) has facilitated development of early warning systems—for extreme heat, for example—which are becoming core responsibilities of many public health departments [97,98,99]. 

In a systematic review of 15 studies in the heat early warning system (HEWS) literature, Toloo et al. found evidence of their cost-effectiveness; these authors noted the importance of careful targeting to the most vulnerable and observed that costs of some adaptive actions (such as running air conditioners) were of concern [100]. Lowe et al. reviewed HEWS in 12 European countries and identified elements for broader dissemination, including addressing shelter and hydration needs of the homeless, and using social media communication [101]. Several reviews noted the importance of using city- or region-specific local definitions of heatwave [100,102]. Li et al. suggested that enhanced use of heat-related morbidity indicators (emergency medical care, hospitalizations) could help optimize HEWS [103]. Noting the changes over time between temperature and health outcomes given observed human acclimatization, Hess and Ebi identified the importance of iterative HEWS that review key parameters every five years or so [97]. 

Early warning systems (EWS) also have been explored for a range of the vector-borne and infectious diseases tracked by public health agencies [98,104,105]. For example, the European Environment and Epidemiology Network has tested with success three prototype case studies of diseases transmitted by mosquitos that hold promise for scaled-up early warning efforts: malaria in Greece; West Nile fever in Southeastern Europe; and dengue fever in Madeira [98]. Efforts are also underway to examine early warning for diseases associated with flooding [106], as well as for cholera and schistosomiasis among others [107,108]. Challenges with infectious disease EWS include complexities of disease transmission, insufficient data, and human mobility [109,110]. 

#### 2.3.2. Link to and Provide Care

Ten years ago, the challenge was seen as planning for emergency medical capacity at all levels in preparation for more frequent and intense weather-related disasters, as well as the need to strengthen mental health capacities for such crises [16]. There has been substantial work on the topic of delivering physical health care to populations affected by climate change, particularly in response to extreme weather events. Rataj et al. found increased burden of injuries and mental health impacts related to extreme weather events in Latin America and Asia, highlighting the need for enhanced public health infrastructure from prevention to treatment [111]. Phua envisioned how national health care systems could be redesigned to respond to climate-related threats by focusing on human resources, facilities and technology and health information systems [112]. Balbus et al. identified five features of successful implementation of sustainable health care systems: (i) an understanding of costs and benefits of climate-sustainability; (ii) an inclusive stakeholder process; (iii) a trained workforce to implement new sustainable systems; (iv) linking sustainability and resilience; and (v) recognizing that community facilities may need to provide shelter and services to neighbors as well as patients in a crisis [113]. Curtis et al. reviewed social care systems in the United Kingdom and stressed that extreme weather event planning should not be limited to emergency response systems [114]. Regarding physical weather disaster preparedness, Boston’s Spaulding Rehabilitation Hospital is an example of a climate-resilient health care facility built based on learning from major coastal storms, with features such as roof-based electrical systems, HVAC redundancy, and natural flood protection berms at its waterfront site [115].

The health sector is also an important contributor to greenhouse gas emissions, and over the decade has become more engaged in quantifying them [116]. Health Care Without Harm has worked to measure carbon footprints of hospitals and advocated reduction of their emissions [117]. The Lancet Countdown has included emissions from the health care field as an indicator [2].

The progression of work on provision of health care services in a changing climate overlaps with findings in other CFES areas (see Research, below). Climate change and mental health research and response is now an active area of work (see for example [118,119]). Balbus et al. raised concern about the low awareness of climate change threats among health care professionals is a barrier to their preparedness [113]. The work by Phua and Curtis et al. highlighted the need to think beyond emergency response systems to practical tools for health care facility preparedness [112,114]. Moving forward, available tools need to be further disseminated and resources made available to implement infrastructure and system improvements. 

#### 2.3.3. Assure Competent Workforce

A decade ago, priority was placed on the need for a basic set of competencies as well as building academic professionals’ skills in—and creating partnerships with—sectors not typically part of the health care and public health curriculum, such as economics, and vulnerability modeling [16]. At that time, two universities mentioned with health and climate change programs were Harvard’s human health and global environmental change, and the University of Wisconsin’s program on humans and the global environment. 

Since 2008, the number of universities and schools of public health with climate change centers, or climate-related education and research missions, has increased to include most major schools of public health. Several of these programs focus on particular aspects of climate change and health, such as Columbia University’s Mailman School which curates a clearinghouse of information on climate change and health educational programs around the world [120]. Efforts to train larger publics include the development of massive open online courses (MOOCs) of a number of universities. Substantial research has called for incorporating climate change into medical school curricula, though implementation challenges remain [121,122,123]. Cross-fertilization with other fields (such as climatology and ecology) to help understand fully the health impacts of climate change, and working with local groups and/or governments to specifically understand the risks to certain geographic areas are suggested strategies for medical work force training [121,123]. Other authors discussed the awareness of public health sector employees already engaged in climate related work; cross-disciplinary partnerships and collaboration within the medical field was stressed as a goal of many health institutions in responding to climate change, but remains in short supply in practice [22,124]. 

#### 2.3.4. Evaluate

Frumkin et al. noted evaluation of preparedness plans and health communication strategies and identified the importance of linking evaluation to surveillance and a well-trained work force [16]. A decade later, the evaluation literature remains sparse but provides useful insights in a broader range of topics, including adaptive interventions, adaptation plans, and economic impacts. For example, Bouzid et al. carried out a systematic review evaluating—among other adaptation interventions—reviews of vector control, water treatment, city greening initiatives, and heatwave early warning systems; these authors noted suggestive evidence of effectiveness for vector control and heatwave early warning; however, the evidence base was considered weak overall [125]. Areas where further research was needed included evaluation of interventions to manage drought, floods, air pollution and food safety [125]. 

Ebi and Otmani del Barrio evaluated health adaptation assessments across a number of low- and middle-income countries and recommended assessments address both shorter-term climate variability and longer-term climate changes [126]. These authors flagged three common needs: (i) indicators for monitoring and evaluation; (ii) training and capacity building; and (iii) sufficient human and financial resources [126]. Panic and Ford reviewed national-level adaptation plans related to infectious disease risks for 14 OECD countries against best practices from peer-reviewed literature; they found that vulnerable populations were seldom considered and implementation was largely sectoral rather than holistic [127]. Economic evaluations of health impacts of climate change-related events in different contexts noted gaps in the types of climate change effects evaluated as well as considerable variability in methods, suggesting a lack of robust economic data to inform decisions on resource allocation for risk reduction [128,129].

### 2.4. Research

A decade ago, research needs included developing the relationship between climate hazards and health outcomes, forecasting of health impacts, identification of population vulnerability, and defining strategies to reduce risk, including their effectiveness and cost [16]. Since then, several studies have attempted to provide an overview of research efforts and needs in the field. In 2009, the WHO identified five areas of research needed to protect health from climate change: (i) assessing health risks; (ii) identifying the most effective interventions; (iii) guiding health promoting mitigation and adaptation in other sectors; (iv) improving decision support with appropriate tools; and (v) estimating the costs of protecting health from climate change [130]. Hosking and Campbell-Lendrum carried out a scoping review to assess the evidence base in these five WHO priority research areas, finding substantial research had been conducted in the association of climate hazards and health outcomes however noting in particular a lack of intervention effectiveness studies [131]. Similarly, in the US context, Sheehan et al. reported the continued need for evaluation of interventions and development of evidence-based best practice [22]. Verner et al. looked at trends in the numbers of publications on climate and health for 1990 to 2014 [132]. Climate and health literature increased substantially over the period but lags compared to other sectors such as transportation and energy. These authors found that malnutrition, non-communicable diseases and mental health in particular were understudied, (as were impacts in low- and middle-income countries) and recommend research capacity-building particularly in the global South [132]. 

## 3. Discussion

In this review, we found evidence of progress in terms of climate change-related public health activities and active use of specific climate and health tools in each of the ten essential services areas (summarized in Table 1). Notably, the evidence of most substantial progress was apparent in the assessment functions of monitoring health status related to climate change hazards, and diagnosing and assessing public health challenges from climate change. In these areas, the public health field is effectively deploying and increasingly refining available tools and methods. We observed a shift from perceived need for enhanced climate-health data and data harmonization (across data sources, as well as spatially and temporally) in 2008, to having relatively robust national and international initiatives and tools to support development of data (international and local climate services, multiple health and climate-related vulnerability indices, enhanced global, national and local surveillance and monitoring). Risk assessment frameworks used in the climate and health field are now increasingly iterative, reflecting uncertainty and the complexity and dynamic nature of climate change. Among the assessment challenges going forward are working out financial sustainability and scaling-up of promising approaches, further data harmonization and refining of methods. 

Progress was also made in the policy development function compared to 2008, although it was more mixed. In terms of mitigation policy, co-benefits to health have attracted substantial research attention, particularly related to air pollution and active transport. However, this has not yet carried over as fully as needed in practice into communication of health benefits to the broader GHG reduction policy debate or policy choices. Regarding adaptation, the public health field is increasingly included in broader climate resilience planning efforts, but there remains a surprising shortage of evaluation studies on adaptation effectiveness (suggesting in part practitioners’ limited time to document or evaluate their work). Meanwhile, our review suggests modest achievements in partnerships, with public health playing a growing role in providing needed data and indicators (e.g., on vulnerability) in multi-disciplinary teams. Public health is poised to take a substantially greater leadership in this domain. However, comparatively less progress seems to have been made in communicating climate and health messaging. Climate health literacy remains low, even among the trusted health care professional population. 

Finally, progress since 2008 in the assurance function was also mixed. In the areas of work force development and intervention evaluation for climate and health related programs, progress over the decade has been disappointingly limited. Uncertainty regarding mandates and resources seems to present practical obstacles to more widespread public health workforce training on climate change, and critical understanding of best practice interventions. However, in the area of enforcing regulations, there was notable forward movement in particular with heat and other early warning systems being developed and overseen by public health authorities. Moreover, in the area of linking to health care, there is evidence of greater hospital and health care system climate risk-awareness and extreme weather preparedness, as well as increased health care system attention toward reducing its own GHG emissions.

Information gathered from our review presented an opportunity to re-consider some aspects of the CFES model. As noted above, we identified several concepts emerging from the literature which are not addressed in the CFES model. Aligning the CFES model with the steps common to the adaptive risk assessment framework increasingly used in climate and health is helpful in illustrating this (Table 1). In particular, we found that three key ideas were missing from the CFES model: First, several of the essential service areas within the assessment and policy development functions overlap with the concept of governance—the institutions, arrangements, funding and mandates to accomplish policy tasks—which has become more prominent in the public health and climate change literatures over the last decade. For example, future needs identified in many of the ten essential services included fuller funding and clearer institutional arrangements. Second, implementation of public health policy and practice is implied rather than explicit in the CFES model, although actual carrying out of activities is a key aspect of both the policy development and the assurance functions. Climate change has brought increased implementation responsibilities to public health agencies (e.g., HEWS), and this is likely to continue. Third, the CFES model inadequately reflects the iterative step of dynamic adjustment, the feedback loop of adaptive learning needed to address complex problems like climate change, and essential in closing the circle from assurance back to assessment. In particular, as evaluation studies and better-quality data improve knowledge, this information will need to be fed into assessments. We propose that these three concepts—governance, implementation and adjustment—be more fully integrated into the CFES model. One way to do so would be to consider them “connector” concepts linking the three core functions.

Our review has several limitations. Our search was limited in scope (focused on review papers, two search engines) and language (English only), and therefore is not an exhaustive examination of all published literature that may be relevant when considering climate change and the ten essential public health service areas. However, we believe that focusing on English-language review papers has given us a representative summary of some of the major themes emerging over the last ten years for the purpose of stock-taking, which was our goal. The nature of the literature itself also presents limitations. Many reviews included in our paper noted that their findings were based upon studies in populations in North America, Europe or Asia, with a corresponding lack of studies in countries in Africa, Latin America and non-China Asia. For example, the review literature on heat early warning systems is largely based on evaluations in the US and Europe due to sparse evaluation studies in other regions. Yet an influential heat warning system in Ahmedabad, India, has recently provided evaluation experience from a low-income heat-vulnerable city [133]—the findings of which will eventually be built into reviews—that will provide relevant lessons for similar contexts. It is a reality of the climate and health field that insufficient research attention has been focused on low- and middle-income populations, and on intervention evaluation more broadly. As noted above, we join other researchers in recommending this be remedied through shifts in research priorities [2,126,131]. The core functions and essential services model was developed in the US based on the American public health system, which inevitably differs from public health systems in other countries and regions. We nevertheless believe that the CFES model has broader generalizability to other settings, and that therefore our review may be relevant not only to the American public health system but also potentially beyond the US public health context.

## 4. Conclusions

Over the past decade, the health impacts of climate change have been keenly felt and will become even more pressing over the decade to come. There has been progress in all of the essential service areas across the core public health functions but in many ways public health has been “playing catch-up” compared to sectors with more experience in climate change planning. To meet the urgent needs of the next decade—decarbonizing the economy and creating resilience for more extreme climate conditions—the public health community must build on this progress. An updated CFES model with strong governance, implementation and dynamic adjustment activities will ensure that the public health field fulfills its potential as a highly valued and proactive partner in climate policy planning and action.

## Figures and Tables

**Table 1:** Applying the public health core functions and essential services model to climate change.

Core Function	Essential Service	Iterative Climate-Health Risk Assessment Framework ^a^	Some Key Available Tools for Health and Climate
**Assessment**	1. Monitor health status 2. Diagnose and investigate health risks	Identify climate impacts and assess vulnerabilities Project disease and impact burden	Climate services for projecting health outcomes Disease surveillance (required syndromic surveillance and health and climate specific monitoring) Adaptive risk assessment for population health risks due to climate hazards Vulnerability assessment, indexes, mapping
**Policy Development**	3. Mobilize partnerships 4. Inform, educate and empower 5. Develop policies and plans	Identify appropriate public heath actions and *governance* ^b^ arrangements Develop health-outcome focused climate action plan	Collaboration, coordination, partnerships across agencies, levels, actors, frameworks Identifying health co-benefits (cost-benefit analysis) Mitigation action planning for health sector Adaptation intervention planning for population health outcomes Communication on health risks of climate hazards, resilience promoting actions, health co-benefits
**Assurance**	6. Enforce laws and regulations 7. Link people to health care 8. Assure competent workforce 9. Evaluate 10. Research	*Implement*^b^ health-focused climate action plan Evaluate impact of interventions and *dynamically adjust* ^b^ based on lessons learned	Vector-borne disease control Water and food safety monitoring Early warning systems (heat, flooding, vector borne disease, air pollution) Nature-based solutions to reduce heat, flood risks Health impact assessment (integration of health outcomes in non-health sectors) Hospital and health care extreme event preparedness Climate change curriculum for public health, medical schools ‘Good practice’ city climate change networks with health focus

^a^ Adapted from US CDC Building Resilience Against Climate Effects Framework (BRACE) and the EU Climate-ADAPT Framework; ^b^ Concepts that enhance the core functions and essential services model.
